# Interbasin water transfers in the United States and Canada

**DOI:** 10.1038/s41597-023-01935-4

**Published:** 2023-01-13

**Authors:** Md. Abu Bakar Siddik, Kerim E. Dickson, James Rising, Benjamin L. Ruddell, Landon T. Marston

**Affiliations:** 1 Department of Civil and Environmental Engineering, Virginia Tech, Blacksburg, VA United States of America; 2 Department of Civil Engineering, Kansas State University, Manhattan, KS United States of America; 3 School of Marine Science and Policy, University of Delaware, Newark, DE United States of America; 4 School of Informatics Computing and Cyber Systems, Northern Arizona University, Flagstaff, AZ United States of America

**Keywords:** Hydrology, Water resources

## Abstract

Interbasin water transfers (IBTs) can have a significant impact on the environment, water availability, and economies within the basins importing and exporting water, as well as basins downstream of these water transfers. The lack of comprehensive data identifying and describing IBTs inhibits understanding of the role IBTs play in supplying water for society, as well as their collective hydrologic impact. We develop three connected datasets inventorying IBTs in the United States and Canada, including their features, geospatial details, and water transfer volumes. We surveyed the academic and gray literature, as well as local, state, and federal water agencies, to collect, process, and verify IBTs in Canada and the United States. Our comprehensive IBT datasets represent all known transfers of untreated water that cross subregion (US) or subdrainage area (CA) boundaries, characterizing a total of 641 IBT projects. The infrastructure-level data made available by these data products can be used to close water budgets, connect water supplies to water use, and better represent human impacts within hydrologic and ecosystem models.

## Background & Summary

Interbasin water transfers convey water from one river basin to another using non-natural means, such as pipelines, aqueducts, or canals. Interbasin transfers (IBTs) can significantly affect water supplies, hydrology, and the environment in both donor and receiving basins^1–3^. Detailed data describing the characteristics of IBTs and their conveyance volumes are needed to close water budgets, connect the place of water diversion to the place of water use, and better represent human influences within hydrologic and ecosystem models. Yet, data describing IBTs are dispersed and incomplete, making analysis of IBTs and their impacts challenging^4^.

This paper describes the development of a comprehensive datasets containing records of conveyance volumes, water use purpose(s), owner/operator, location, and other infrastructure details for IBTs in the United States and Canada. While the US Army Corps of Engineers maintains a National Levee Dataset^5^ and a National Inventory of Dams^6^ for the United States, there is not a similar comprehensive, federally maintained database of IBTs. Our IBT dataset builds on previous efforts to inventory and map IBTs in the United States^7–9^ and Canada^10,11^. Previous US and Canadian IBT datasets provided an incomplete (and now outdated) sampling of IBTs^8–11^ or over-represent IBTs by including inconsequential drainage ditches, unverified conveyance infrastructure, and double counting multiple instances of water transfers within the same IBT project as separate IBTs^7^. Our datasets are unique, however, in their completeness, detail, and inclusion of sub-annual conveyance volumes.

Working with local, state, and federal partners, we have identified all known transfers of untreated water across 4-digit hydrologic unit code (HUC4) subregions in the US and sub-drainage areas in Canada. A new data standard, named the Interbasin Transfer Database Standard Version 1.0. (IBTDS 1.0), is used to organize and standardize the data for query. Local officials were asked to verify all entries within our dataset. The final data products have undergone multiple rounds of internal and external quality assurance/quality control (QA/QC).

**Fig. 1:**
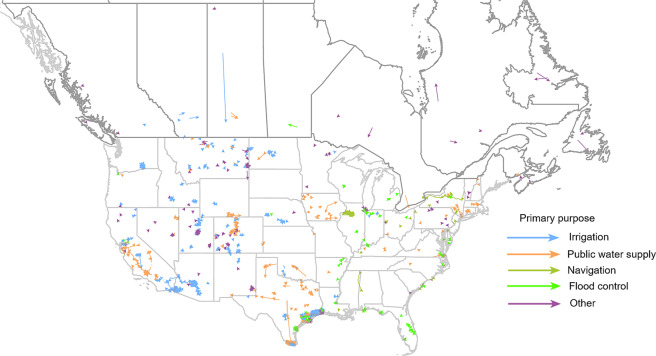
Mapping of United States and Canadian interbasin water transfers by primary purpose.

**Fig. 2:**
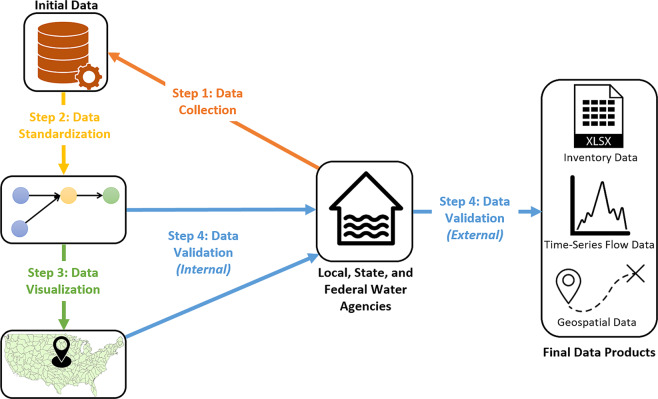
Summary of dataset development. First, data were collected from local, state, and federal agencies, as well as the academic and gray literatures. Second, primary data was standardized using our data standard, IBTDS 1.0. Third, data was visualized in ArcGIS Online. Fourth, all data was reviewed and verified by the research team and local, state, and federal officials.

This paper documents three inter-related IBT datasets using the IBTDS 1.0 for US and Canadian IBTs: (i) tabular inventory data detailing the water source, place of use, owner/operator, project name, use purpose(s), infrastructure properties, and other pertinent details of each IBT; (ii) time-series records of water transfer volumes for select IBTs in the US; and (iii) geospatial data showing the location, flow path, and other features of each IBT. Figure 1 shows the location and purpose of all IBT projects within our datasets. A schematic overview of the development of these data products and a description of the three dataset contents are found in Fig. 2 and Table 1, respectively.

**Table 1:** Overview of the three interbasin water transfer (IBT) data products.

Data Products	Description	Data Format	Reference
IBT Inventory Data	Tabular data detailing the water source, place of use, owner/operator, project name, use purpose(s), infrastructure properties, and other pertinent details of each IBT.	Excel Spreadsheet (XLSX)	^21^
IBT Time-Series Flow Data	Tabular time-series records of daily water transfer volumes for select IBTs in the US.	Excel Spreadsheet (XLSX)	^21^
IBT Geospatial Data	Geospatial data showing the location, flow direction/path, and features of each IBT.	Esri Shapefile (SHP)	^21^

## Methods

**Fig. 3:**
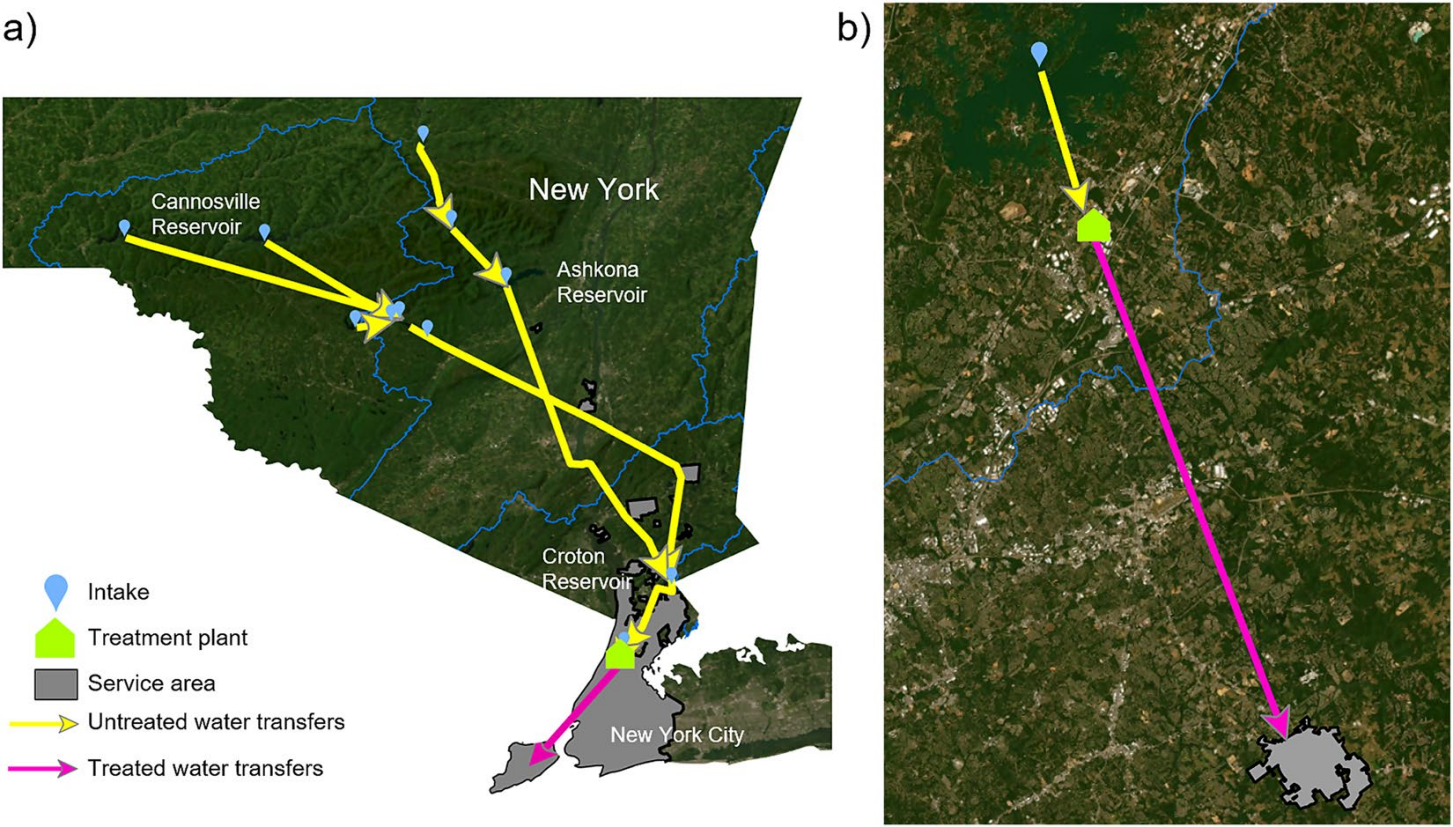
Examples of potential interbasin transfers of raw water (**a**) and treated water (**b**). Raw water transfers are represented by yellow lines, while a treated water transfer is represented by a magenta line. If raw water crosses a subregion boundary (blue lines), it is included in our dataset, as is the case for the Schoharie and Delaware Aqueducts that bring raw water for New York City public water supply (**a**). If only treated water crosses a subregion boundary, as is the case for Gwinnett County’s public water supply system in Georgia (**b**), then it is not included within our IBT datasets.

Interbasin water transfers have been defined many ways within the literature^12–14^ and by government agencies. For this study, we define an IBT as a human-mediated movement of surface water or groundwater from one sub-drainage area or subregion (HUC4) to another sub-drainage area or subregion through man-made or artificial pathways (e.g., canals, pipelines, aqueducts). Subregion^15^ and sub-drainage^16^ boundaries come from the United States Geological Survey (USGS) and Natural Resources Canada, respectively. We further narrow our IBT definition to exclude the transfer of treated water and wastewater due to the lack of data describing complex municipal water and wastewater distribution systems across Canada and the US. The movement of untreated (or “raw”) water between the intake location of a water distribution system and the water treatment facility is deemed an IBT if it traverses a basin boundary (i.e., sub-drainage or subregion boundary; e.g., Fig. 3a); however, if water within the distribution system crosses a basin boundary *after* treatment, we do not include this instance within our IBT datasets (Fig. 3b). We have also removed inconsequential drainage ditches that drain less than 0.5 square kilometers. Such drainage ditches constituted a significant fraction of previous US IBT datasets^7^, even though they have a negligible hydrologic, ecological, or societal impact.

The creation of our IBT data products involved four steps: i) data collection, ii) data standardization, iii) data visualization, and iv) data validation. The first three steps are described in this section (Methods), while data validation is described within the ‘Technical Validation’ section.

### Data collection

To create a national IBT dataset, we started with *potential* IBTs identified by Dickson and Dzombak^7^. Dickson and Dzombak extracted all artificial flow paths that crossed subregion boundaries from the USGS National Hydrography Dataset (NHD). These IBTs were not verified and lacked descriptive details, such as water use purpose or transfer volume. Furthermore, the number of IBTs reported by Dickson and Dzombak is artificially large since it counts each instance a conveyance structure crosses a basin boundary as an individual IBT, even if it is part of one larger IBT project (e.g., Central Arizona Project). These records were paired with older IBT datasets produced by USGS^8,9^. Together, these datasets represent the most complete US IBT datasets to date. We filtered out records from the combined datasets that did not meet our IBT definition, were duplicates, or were verified as being either decommissioned or erroneous. We also connected flowlines that are part of the same IBT project.

Next, we searched state and federal reports, data repositories, and websites for data describing the location, properties, and flow volumes of IBTs. Findings from these searches allowed us to remove erroneous records within our current dataset, as well as add new IBTs that were not captured by previous datasets. Mostly, though, our review of government records allowed us to confirm IBT records and to provide more complete documentation of already identified IBTs. Websites for federal agencies that have a role in building, administering, or maintaining records on IBTs, such as the USGS, US Bureau of Reclamation (USBR), US Army Corps of Engineers (USACE), and the Environmental Protection Agency (EPA), were searched for relevant records. Approval by USACE is required when building across a navigable waterway, which is sometimes required for IBTs. Much of the major federal water supply infrastructure in the Western US, including IBTs, were built and are currently operated by USBR. The EPA has records related to water distribution systems^17^, including water intake and treatment locations, which were used to identify IBT locations. The USGS gauge network reports time-series records for 79 IBTs. Relevant state websites for IBT data collection were identified through the survey of state-level water data platforms developed by Josset *et al*.^18^.

After reviewing the scientific literature and publicly available government reports, data repositories, and websites, we contacted federal, state, and local representatives for additional data records and to verify our existing records. Federal employees at USGS and USBR reviewed and provided additional records for our initial IBT dataset. The USGS Water Use Science Project regularly collects water use and water infrastructure data from states. The USGS Water Use team helped us identify the state agency and contact person that would most likely maintain IBT data for each state.

We sent IBT data requests to each state via email and phone calls. In cases where these attempts were unsuccessful, we filed an Open Records Act or Freedom of Information Act (FOIA) request to collect any remaining data we were missing. In cases where neither federal or state agencies maintained the data we sought, we contacted IBT operators directly. Direct contact with IBT operators was primarily done when collecting time-series flow data for irrigation districts and municipal water suppliers.

Canadian IBT data were collected from an Access to Information Act records request. The Environment and Climate Change Canada (formerly, Environment Canada) had maintained records of IBTs throughout Canada until 2011. Several reports published by Environment Canada researchers^10,11^ document Canadian IBTs and their properties. These reports highlight select IBTs but do not provide complete IBT records. Our Access to Information Act request provided us an unpublished report and associated data from 2004 that described the full collection of IBTs in Canada.

Data were collected between August 2019 and June 2022. Our data products reflect the most up-to-date data held by primary data collectors on the date of our request. The date each IBT entry was collected is reported in the IBT Inventory Dataset. We collected all time-series flow data available for each IBT, with some records going back as far as 1901.

### Data standardization

The data we collected were in a variety of file formats and data types. We created a data standard, which we named the Interbasin Transfer Database Standard Version 1.0. (IBTDS 1.0), to provide a consistent way of representing and defining data for all IBTs. The standardized IBT Inventory Dataset follows a node-link structure. Nodes represent places of water diversion, water use, or change in flow (e.g., reservoir, channel junction). Links represent conveyance infrastructure or natural waterways that connect two or more nodes within an IBT project. Unique link identifiers (Link ID) connect two or more unique node identifiers (Node ID). One or more links constitute an IBT project. The owner/operator of each IBT project, as well as the year the IBT project was commissioned and decommissioned (if applicable), is reported within the IBT Inventory Dataset.

Geospatial details are reported for each IBT project in the IBT Inventory Dataset and the IBT Geospatial Dataset. We obtained the precise latitude and longitude of each node using the various data sources noted previously, as well as visual inspection of high-resolution aerial imagery from Google Earth and Esri’s World Imagery layer. Precise geospatial information is reflected in the IBT Geospatial Dataset. The IBT Inventory Dataset lists the hydrologic and geopolitical boundaries that contain each node. For the US, the state and county name and the Federal Information Processing System (FIPS) Code is also provided for each node. Likewise, the province and Census Geographic Unit is given for each node in Canada. The IBT project name (e.g., Heron Bayou Drainage Ditch, Hennepin Canal) associated with each node and link segment is also reported.

**Fig. 4:**
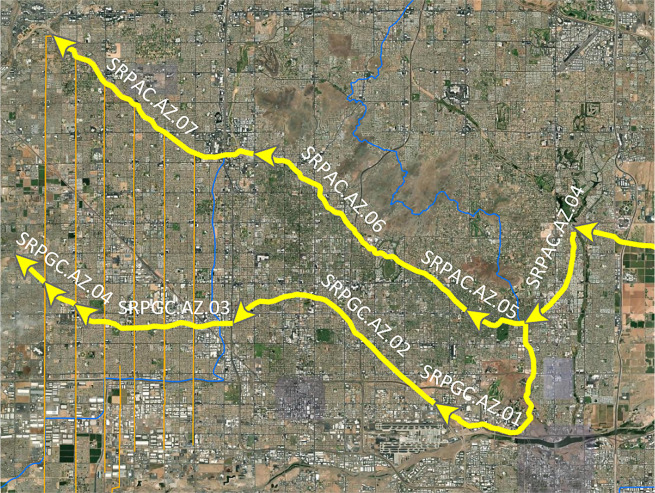
An example of an interbasin water transfer project in Arizona with major (yellow) and minor (orange) project components. The thick yellow lines represent primary components of the project that are recorded in our dataset and assigned a Link ID (white text label). The thin orange lines represent secondary or tertiary canals or ditches that are small relative to the main (yellow) project segments and are therefore not represented in our dataset. The blue lines represent HUC4 subregion boundaries.

As is often the case with irrigation and drainage IBT projects, there are sometimes several relatively small, adjacent diversions/ditches along an IBT project. We focus on capturing the main components of the IBT, instead of representing dozens or even hundreds of connected small ditches that divert or collect water along the IBT project. Nonetheless, when the collective impact of these small water diversions or inputs may noticeably change IBT flows, we depict these small ditches together as a representative two-node pair connected by a link segment. One of the nodes represents approximately the middle of where these small ditches intersect with the main IBT channel. The other node is the approximate centroid of water users served by or areas drained by these small ditches. If one of the secondary channels is large relative to the main channel (i.e., ability to divert more than ~25% of the main channel flow based on channel top width or flow records), it is recorded with its own Node ID and Link ID (Fig. 4). Likewise, if a secondary channel has an official name granted by a government agency or its owner/operator, we also record this segment with its own Node IDs and Link ID(s).

We record the primary, secondary, and tertiary purpose of each IBT project and these purposes are the same for all links within the IBT project. One of “water supply - public supply”, “water supply - irrigation”, “flood control”, “navigation”, “waste discharge”, “environmental flows”, “energy - hydroelectric”, “energy - thermoelectric”, “energy - mining”, “other”, or “unknown” is assigned to each IBT project based on online records, design documents, reports, and/or personal correspondence with local, state, or federal officials. Link infrastructural properties, such as whether the link is a lined canal, unlined canal, pipe/tunnel, or other structure, are recorded for each link segment.

The average water transfer rate (m^3^/d) is reported for each link segment where this information is known. The average water transfer rate only represents flows for the identified link segment, not necessarily the entire IBT project since upstream/downstream diversions and inputs may mean flow rates are different in different portions of the project. The average water transfer rate is converted from the units provided to us but is otherwise left unchanged. The primary data records are often unclear or do not specify the time period used to estimate average water transfer rates. The IBT Inventory Dataset reports whether time-series data is available for each Link ID in the IBT Time-Series Flow Dataset.

The IBTDS 1.0 data standard was also applied to the IBT Time-Series Flow Dataset. The unique Link ID identifying the location where the transferred flow rate was measured is recorded for each time-series entry, relating the time-series data records to the IBT Inventory Dataset. The recorded flow rate only represents water transfer rates for the given link segment where the measurement was made, not necessarily the entire IBT project. Time-series data describing IBT flow rates were recorded at various temporal resolutions, ranging from instantaneous gauge readings every 15 minutes to average annual records. The standardized time-series dataset converted all reported water transfers to a common measurement unit (m^3^) and temporal resolution (day). When available, a web link to the original data source is published with the standardized data. The original timestep which the data was collected is also reported for each entry.

In a few instances, there is more than one flow measurement for a link segment. Measurements are typically reported by different agencies and the measurements do not always align perfectly, either in their quantity or frequency of their reporting. Unless one of the records is known to be erroneous or of inferior quality, both sets of records are standardized and reported. For example, USBR reports monthly water transfer volumes along the Central Arizona Project (Link ID: CAP.AZ.01), while USGS reports daily water transfer volumes for the same link segment.

### Data visualization

We provide an online visualization of the IBT Geospatial Dataset using ArcGIS Online (https://virginiatech.maps.arcgis.com/apps/mapviewer/index.html?webmap=b2cfac9b70ea44e4938734da0b1a7c8e), which is also summarized in Fig. 1. Every IBT node and link segment in the IBT Inventory Dataset is included. An arrowhead at the end of a link segment depicts the flow direction of transferred water. Link segments imported into ArcGIS Online were initially represented as a straight path between connected nodes. When the IBT flowpath was visible from aerial imagery or the flowpath was available from existing sources (e.g., NHD or detailed engineering drawings), the exact path of transferred water was mapped; otherwise, the flowpath remained a straight line between connected nodes.

State and federal agencies restricted some of the data we are able to share publicly. Specifically, we are not permitted to reveal the exact water intake and treatment locations of some public water suppliers. Instead of mapping the precise latitude and longitude of points of diversion, points of flow change, and points of use like with other IBTs, IBTs whose primary purpose is public water supply are depicted as a straight line connecting the centroids of subwatersheds (HUC12) where the IBT node is located.

## Data Records

The three IBT datasets (i.e., inventory, time-series, and geospatial) were archived with HydroShare^19^, a data sharing platform operated by the Consortium of Universities for the Advancement of Hydrologic Science Inc. (CUAHSI) (www.HydroShare.org). Each dataset has been assigned a unique digital object identifier (DOI), each of which is provided in Table 1. Data is stored as either an Excel spreadsheet (XLSX; IBT Inventory and IBT Time-Series Flow Datasets) or an Esri shapefile (SHP; IBT Geospatial Dataset). IBT time-series flow data for each state is maintained in its own spreadsheet, with each spreadsheet tab representing flow measurements for a particular IBT link segment.

The IBT Inventory Dataset contains 2480 nodes and 1910 links representing 641 unique IBT projects. There are 615 IBT projects in the US and 26 IBT projects in Canada. The IBT Time-Series Flow Dataset contains 165 time-series records for 134 IBTs in the US. Some IBT projects do not have time-series data but may have an annual average flow estimate. There are 295 IBT projects in Canada and the US with either time-series data or annual flow rate estimates. The average annual water transfer volume of the 269 US IBTs with flowrate data is 46.4 km3. However, there are a handful of large IBT projects primarily used for non-consumptive purposes, such as flood management and navigation. The primary purpose of all 26 of Canada’s IBT projects with flow data is for hydroelectricity generation or flood management, which are non-consumptive uses. These Canada IBTs transfer 85.3 km3 of water annually. There are 192 IBT projects in the US with flow records whose primary purpose is public water supply or irrigation. These projects transfer 24.9 km3 of water, which amounts to 19% of these two sectors reported surface freshwater withdrawals^20^. A summary of IBT records by HUC2 Region (US) and Drainage Area (CA) is presented in Table 2 and Table 3, respectively.

**Table 2:** Summary of interbasin water transfers by primary purpose within each United States’ HUC2 Region. The number of interbasin transfer projects with flow data is reported in the last two columns.

HUC2 Region	Number of IBT projects originating in HUC2 with primary purpose:						Number of IBT projects with flow data	
	Irrigation	Public supply	Navigation	Flood control	Other	Total	Average annual flow data	Time-series data
New England Region (01)	0	11	0	0	2	13	9	3
Mid-Atlantic Region (02)	0	8	5	7	3	23	14	4
South Atlantic-Gulf Region (03)	0	6	8	20	1	35	10	8
Great Lakes Region (04)	1	18	3	8	6	36	9	7
Ohio Region (05)	1	8	5	9	4	27	10	6
Tennessee Region (06)	0	1	1	0	0	2	1	1
Upper Mississippi Region (07)	2	8	2	3	1	16	8	1
Lower Mississippi Region (08)	2	4	2	7	2	17	2	1
Souris-Red-Rainy Region (09)	1	0	0	0	1	2	1	0
Missouri Region (10)	45	29	0	1	20	95	72	14
Arkansas-White-Red Region (11)	3	26	0	2	1	32	4	2
Texas-Gulf Region (12)	34	46	0	15	23	118	19	4
Rio Grande Region (13)	16	19	0	0	6	41	6	5
Upper Colorado Region (14)	22	22	0	0	20	64	50	48
Lower Colorado Region (15)	20	3	0	0	2	25	17	14
Great Basin Region (16)	5	1	0	0	8	14	10	3
Pacific Northwest Region (17)	19	2	0	1	3	25	10	5
California Region (18)	10	14	0	1	5	30	17	8

**Table 3:** Summary of interbasin water transfers by primary purpose within each Drainage Area in Canada.

Drainage Area	Number of IBT projects originating in Drainage Area with primary purpose:						Number of IBT projects with flow data	
	Irrigation	Public supply	Navigation	Flood control	Other	Total	Average annual flow data	Time-series data
Maritime Provinces Drainage Area (01)	0	1	0	0	2	3	3	0
St. Lawrence Drainage Area (02)	0	1	0	0	4	5	5	0
Northern Québec and Labrador Drainage Area (03)	0	0	0	0	4	4	4	0
Southwestern Hudson Bay Drainage Area (04)	0	0	0	0	2	2	2	0
Nelson River Drainage Area (05)	3	2	0	1	0	6	6	0
Western and Northern Hudson Bay Drainage Area (06)	1	0	0	0	0	1	1	0
Great Slave Lake Drainage Area (07)	0	0	0	0	1	1	1	0
Pacific Drainage Area (08)	1	0	0	0	3	4	4	0
Yukon River Drainage Area (09)	0	0	0	0	0	0	0	0
Arctic Drainage Area (10)	0	0	0	0	0	0	0	0
Mississippi River Drainage Area (11)	0	0	0	0	0	0	0	0

The number of interbasin transfer projects with flow data is reported.

Data records in the IBT Inventory Dataset and IBT Time-Series Flow Dataset are summarized in Tables 4–5 and Table 6, respectively. Table 4 lists the node data features and a description of the feature properties. Table 5 lists the link data features and a description of the feature properties. Table 6 details the information and data features recorded for each time-series record of water transfer flow rates.

**Table 4:** IBT Inventory Dataset node features.

Node Features	Feature Description
Node ID	A unique 7-character string assigned to each node. The first two characters are the state abbreviation where the node is located, followed by a sequential numbering. The last character specifies whether the node represents a natural (*N*; e.g., water diverted from or delivered to a river) or anthropogenic (*A;* e.g., water delivered directly to a water user or transferred from a different artificial channel segment) entity.
Node Name	For source nodes, the name of the source of the water; for destination nodes, the name of the final use; for intermediate nodes, a descriptive name. Example: Lower Fork Reservoir.
Node Type	One of “Source”, “Intermediate”, or “Destination”. Source identifies the water body that the transferred water originated. Intermediate means a location along the IBT project where the flow of water is significantly altered in some way, such as water storage, major channel junctions, intermediate diversions, or additional inflows. Destination refers to where the transferred water ultimately flows, drains, or is used.
IBT Project	The name of the IBT project the node belongs to. Example: Central Arizona Project.
HUC12/WSCSSDA Code	The 12-digit Hydrologic Unit Code (HUC12; for US) or the Water Survey of Canada Sub-Sub-Drainage Area code (for Canada) where the node is located.
HUC12/WSCSSDA Name	The subwatershed name (HUC12; for US) or the Water Survey of Canada Sub-Sub-Drainage Area name (for Canada) where the node is located.
HUC4/WSCSDA Code	The 4-digit Hydrologic Unit Code (HUC4; for US) or the Water Survey of Canada Sub-Drainage Area code (for Canada) where the node is located.
HUC4/WSCSDA Name	The subregion name (HUC4; for US) or the Water Survey of Canada Sub-Drainage Area name (for Canada) where the node is located.
FIPS/CDUID	The 5-digit Federal Information Processing Standard (FIPS) code (for US) or the 4-digit census division unique identifier (for Canada) where the node is located.
County/Census Geographic Units	The county name (for US) or census division name (for Canada) where the node is located.
State/Province	A 2-letter state code (for US) or province code (for Canada) where the node is located.
Country	A 2-letter country code (US or CA) where each node is located.

**Table 5:** IBT Inventory Dataset link features.

Link Features	Feature Description
Link ID	A unique string identifier for each conveyance (e.g., channel, ditch, canal, tunnel, pipe, stream) segment connecting two nodes. A common 2–5 letter prefix (often a simple abbreviation of the formal IBT project name) identifies the IBT project; all link segments within an IBT project share the same prefix. The prefix is followed by a period and the state abbreviation for the state in which the IBT originates. A unique two-digit sequential number for each link segment within the IBT project ends the string. *Example: The Link ID of the fifth link segment in the Central Arizona Project (CAP) in Arizona is CAP.AZ.05*
State/Province of Origin	A 2-letter state code (for US) or province code (for Canada) where the link segment originated.
Country	A 2-letter country code where the link segment originated.
Node ID-Start	The Node ID associated with the node where the link originates.
Node ID-End	The Node ID associated with the node where the link terminates.
IBT Project	The name of the IBT project the link belongs to. Example: Central Arizona Project.
IBT Project Primary Purpose	One of “water supply - public supply”, “water supply - irrigation”, “flood control”, “navigation”, “waste discharge”, “environmental flows”, “energy - hydroelectric”, “energy - thermoelectric”, “energy - mining”, “other”, or “unknown”.
IBT Project Secondary Purpose	One of “water supply - public supply”, “water supply - irrigation”, “flood control”, “navigation”, “waste discharge”, “environmental flows”, “energy - hydroelectric”, “energy - thermoelectric”, “energy - mining”, “other”, or “unknown”.
IBT Project Tertiary Purpose	One of “water supply - public supply”, “water supply - irrigation”, “flood control”, “navigation”, “waste discharge”, “environmental flows”, “energy - hydroelectric”, “energy - thermoelectric”, “energy - mining”, “other”, or “unknown”.
Owner/Operator	Who owns and/or operates the IBT project. Example: US Bureau of Reclamation
Link Type	One of “pipe/tunnel”, “canal - lined”, “canal - unlined”, “canal - mixed/unknown”, “facility”, or “unknown”. An example of a ‘facility’ would be when the water is transferred through a complex system of pipes or canals, such as an irrigation district or public utility distribution system.
Year IBT Project Commissioned	Year when the IBT project became operational.
Year IBT Project Decommissioned	Year when the IBT project stopped long-term operation (if applicable).
Time-series Data (Available/Not Available)	Specifies whether a metered flow record is available for the link segment. One of “Available” or “Not Available”.
Average Water Transfer Rate (m³/d)	The average amount of water flowing through the link segment during its operation in units of cubic meters per day (m³/d). The average daily water transfer rate is calculated over the time period the original data was provided. Measurements of transfer flow volume, discharge, or depth relate to a specific link and represent where the measurement was made, not necessarily the flow through the entire IBT project.
IBT Contact Person	The name and email of the person that provided and/or verified the data about the IBT.
IBT Contact Agency	The agency or organization the person that provided and/or verified the data about the IBT is affiliated.
Date Recorded	Date when the data was recorded, as YYYY-MM-DD.

**Table 6:** IBT Time-Series Flow Dataset features and information.

Feature/Information	Description
Link ID	Identifier given to the conveyance where water transfers are measured or estimated. The Link ID is the same as the identifier used in the IBT Inventory Dataset.
Data Collection Frequency	How often flow rate measurements were made (e.g., hourly, daily, monthly, annually).
Original Data Source	Web link of original data source (if applicable).
Transfer Date	Day water transfer took place, as MM/DD/YYYY.
Transfer Flow Rate (m^3^/d)	The daily volume of water transferred, in m^3^/d.

## Technical Validation

Data went through three rounds of quality assurance and quality control before publication. First, all data entries were internally reviewed by our research team. Standardized data were compared against the primary data, as well as secondary data sources (e.g., articles, reports, or websites that corroborate the primary data), to ensure data entries were correct. Each entry was coded and reviewed for accuracy and completeness by one team member. Entries were then reviewed by one or more other team members. Mapping of IBTs allowed us to visually identify any geospatial records that were misreported or coded incorrectly. Where possible, we searched for visual confirmation of the IBT geospatial records from high-resolution aerial imagery. Second, data for each IBT were provided to state and/or local officials for review and verification. Third, the three data products were reviewed by multiple USGS and USBR staff. The federal agency review focused on potential mislabeling of features, deviations from our data standards, and erroneous records.

## Data Availability

No code was used in dataset development.
